# Factors Affecting the Incidence of Double Ovulations in Lactating Dairy Cows: Estrous Cycle Length

**DOI:** 10.3390/ani15203000

**Published:** 2025-10-16

**Authors:** Fernando López-Gatius, Irina Garcia-Ispierto

**Affiliations:** 1 Agrotecnio Centre, 25198 Lleida, Spain; irina.garcia@udl.cat; 2 Transfer in Bovine Reproduction SLu, 22300 Barbastro, Spain; 3 Department of Animal Science, University of Lleida, 25198 Lleida, Spain

**Keywords:** animal welfare, dark–light cycles, follicular waves, multiple ovulations, standardized transmitting ability, twinning

## Abstract

**Simple Summary:**

Double ovulations and subsequent twin pregnancies impair the health and welfare of a dairy cow, such that dairy herd profitability decreases in parallel with an increase in the twinning rate. Genomic prediction values for twin pregnancies derived from twin births or advanced twin abortions can help identify the risk of both double ovulations and early twin pregnancies. Independently of genomic predictions, however, a negative photoperiod, in other words, decreasing day length, which in our region extends from 22 June to 21 December, is a strong factor favoring multiple ovulations and multiple pregnancies. The objective of this study was to investigate factors affecting the incidence of double ovulations in high-producing primiparous dairy cows, with special emphasis on estrous cycle length as a possible influencing factor. Our results indicated that the incidence of lengthened cycles was greater during the negative compared to positive photoperiod; double ovulation rates were higher in lengthened cycles compared to normal ones; genomic prediction values for twin pregnancies were associated with the incidence of lengthened cycles; and the length of the photoperiod and genomic prediction values were both, independently of each other, related to the incidence of double ovulations.

**Abstract:**

This study was designed to identify factors, including estrous cycle length, affecting the incidence of double ovulations in dairy cows. The study population comprised 748 primiparous cows undergoing spontaneous estrous cycles that had undergone their first postpartum artificial insemination following the second observed estrus. A subset of 341 cows with inter-estrus intervals (IEIs) of 18 to 30 days were selected to investigate the impacts of IEI, which was classified as normal (18–23 days) or lengthened (24–30 days). The odds ratio (OR) for double ovulations was 0.82 (*p* < 0.0001) for each unit increase in genomic prediction values for twin pregnancies, 2.3 (*p* < 0.0001) for cows inseminated during the negative photoperiod, compared to cows inseminated during the positive photoperiod, and 2.8 (*p* = 0.02) for cows with lengthened cycles, compared to cows with normal cycles. The OR for lengthened IEIs was 0.91 (*p* = 0.001) for each unit increase in genomic prediction value, and 4.4 (*p* < 0.0001) for cows inseminated during the negative photoperiod. In conclusion, lengthened estrous cycles were associated with double ovulations, genomic prediction values were able to identify the risk of lengthened cycles, and the negative photoperiod was found to favor both lengthened cycles and double ovulations.

## 1. Introduction

The concept of two follicular waves per cycle was proposed in gross histological studies conducted in cattle in 1960 [1,2]. After 28 years of conflicting reports, this two-wave hypothesis was finally confirmed in 1988 based on ultrasound technology [3,4,5,6,7]. A follicular wave observed two or three times during the bovine estrous cycle involves the synchronous growth of a group of antral follicles of which one (dominant follicle) continues to grow, whereas the others (subordinate follicles) enter a process of atresia. Thus, follicular dynamics during an estrous cycle is marked by the growth of one or two nonovulatory follicles of ovulatory size that undergo atresia before the maturation of the final ovulatory follicle [3,4,5,6,7]. Cycles with one or four waves occur at a low incidence [4,8,9,10]. There is also evidence that the production of follicular waves is a common strategy employed by several, if not all, monovular mammalian species to produce a single ovulatory follicle [11,12,13,14]. However, aspects of the mechanisms involved in the development of follicular waves remain to be clarified in cattle [15,16,17,18]. Lactating dairy cows tend to have two follicle waves per 18–23-day cycle, whereas three waves are observed in cycles of 24 or more days [8]. In fact, it has been extensively described that three-wave inter-ovulatory intervals are significantly longer than two-wave intervals [3,5,8,9,10,19,20]. Therefore, we could consider that inter-estrus intervals (IEIs) of 24 days or more are likely to include a higher proportion of cycles with three follicular waves. Of course, a cycle exceeding 30 days may be more indicative of a double IEI, following failure to detect estrus, than a lengthened cycle [21,22].

Although twinning is of great interest to beef cattle farmers [23,24,25], both twin pregnancies and twin births compromise the health and wellbeing of dairy cows [26,27,28,29,30]. Consequently, twin pregnancies are undesirable as they lead to significant economic losses for dairy herds [31,32,33]. As genetic correlations between twinning rates and other traits are very low [34,35,36,37,38], it is of interest to identify genomic tools to reduce the twin pregnancy rate. Most twin births (around 90%) are dizygotic, resulting from the ovulation of two codominant follicles from one or both ovaries [23,28,39,40,41,42,43,44]. This situation determines a double ovulation rate that is much higher than its corresponding twin birth rate [39,40]. The incidence of double ovulations and twin births rises with increasing cow age and a negative photoperiod. In pregnant cows in their third lactation or more, multiple ovulations and early multiple pregnancies (28–34 days of gestation) have been found to exceed 50% (2693/5373) and 25% (1395/5373), respectively, whereas rates of multiple ovulations and multiple pregnancies were significantly lower for the positive (increasing day length: 45% and 23.8%, respectively) than negative photoperiod (decreasing day length: 54.7% and 27.9%, respectively) [40]. In two recent studies, genomic prediction values derived mostly from twin births [35] served to identify the risk of double ovulations and early twin pregnancies (28–34 days of pregnancy) at the herd level [45,46]. In a study population of 775 primiparous cows in their first postpartum pregnancy, independently of genomic prediction values, the odds ratio for twin pregnancies was 3.5 for cows becoming pregnant during the negative photoperiod, compared to the remaining cows that became pregnant during the positive photoperiod [45].

Regarding the impacts of factors related to follicular waves, compared to the dominant follicle of the second wave, the dominant follicle of the first wave is more active in terms of growth, blood flow in the follicular wall and steroidogenic capacity, and also in the activity of the resulting corpus luteum (CL) following its induced ovulation [47,48]. Follicular codominance, defined as the presence of two follicles measuring at least 10 mm [49,50,51,52,53], has been observed during the first wave with an incidence up to 35% in heifers [49] and 40% in cows [52], with codominance being much lower (4%) in the second wave [49]. In lactating Holstein/Friesian cows showing spontaneous estrous cycles, double ovulations were observed more frequently in cows showing three (10/35: 28.6%) and four or more (2/5: 40%) follicular waves per cycle than in cows with two-wave estrous cycles (2/118: 0.3%) [10].

Using genomic prediction values for twin pregnancy as an independent variable to examine factors influencing double ovulation rates, the aims of this retrospective cohort observational study were to: (a) assess cow and environmental factors affecting the incidence of double ovulations in primiparous lactating dairy cows undergoing spontaneous estrous cycles, with special emphasis on the possible influence of estrous cycle length; and (b) examine the possible influence of photoperiod on the incidence of lengthened estrous cycles. Our hypotheses were that double ovulation rates would be favored by IEIs of 24 days or more and that such lengthened estrous cycles would predominate during the negative photoperiod.

## 2. Materials and Methods

### 2.1. Cows and Herd Management

The data used for this study were derived from a Holstein dairy herd raised in the Ebro Valley, north-eastern Spain (latitude 41.13 N, longitude −2.4 E). During the study period (from May 2023 to June 2024), the mean number of lactating cows in the herd was 4402, and mean annual milk production was 17,228 kg per lactating cow. Cows were milked three times daily, and with an annual herd expansion rate of 8% by using their own heifer replacement, the culling rate during the study period was 23.4%. Cows were grouped according to age (primiparous cows, secundiparous cows, or cows in their third lactation or higher) and fed complete rations which were the same from Day 30 postpartum to dry off. Walking activity values were obtained using pedometers at the milking parlor (three times daily). When estrus was detected by the pedometer system (AfiFarm System; Afikim, Israel), this was confirmed by rectal palpation before artificial insemination (AI). Only cows with the uterus highly turgid and contractile to the touch and with vaginal discharges copious, fluid, and transparent obtained following uterine massage were inseminated. Data were automatically analyzed using a herd management computer program. Herd management included the use of fans in cubicles, and feeding and milking areas. Water sprinklers were placed in the feeding area and waiting room of the milking parlor, with water spray directed towards the cows. Fans and water sprinklers throughout the cubicle and feeding areas were automatically activated when temperatures reached approximately 23 °C and 25 °C, respectively, whereas in the waiting room of the milking parlor, fans were activated at approximately 10 °C and water sprinklers at 18 °C [54]. The herd was maintained on a weekly reproductive health program based on ultrasonography, as described elsewhere [40,44,45,46]. As reference, the conception rate in parous cows of 47.9% following the first AI during the study period was employed. The rates of multiple ovulations and twin pregnancy in lactating cows were 23.4% and 11%, respectively.

Only primiparous cows delivering female singletons, derived from sex-sorted semen, undergoing postpartum spontaneous estrous cycles that experienced their first postpartum AI following the second observed estrus and before 90 days in milk (DIM) were enrolled in the study. As previous twinning is a strong factor favoring twin pregnancies [28,55] and the twinning rate for primiparous cows was very low (0.4%: 5/1379), cows delivering twins were not included. Cows received no type of hormone treatment to avoid a possible effect of hormone protocols for fixed-time AI on the risk of double ovulation [45,46,56]. The threshold value of 90 DIM proved to be strongly useful in previous studies on factors influencing double ovulation [40,57]. The odds ratio for multiple ovulations was 4.3 for cows becoming pregnant ≥ 90 DIM, compared to the remaining cows becoming pregnant < 90 DIM [40]. Only healthy cows were included in the study, as indicated by a body condition score of 2–3.5 on a scale of 1 to 5 [58]. These cows produced more than 30 kg of milk per day at the time of AI and were free of clinical signs of disease during the study period (days 0 to 34 post-AI). All animals were reared within the herd.

### 2.2. Ultrasound Exams and Study Population

All gynecological exams were performed by transrectal ultrasonography by the same operator using a portable B-mode ultrasound scanner equipped with a 5–10 MHz transducer (E.I. Medical IBEX LITE; E.I. Medical Imaging, Loveland, CO, USA). Each ovary was scanned in several planes by moving the transducer along the surface to identify luteal structures. The size, number, and location of CL were recorded. Ovulation was assessed by the presence of at least one mature CL of at least 12 mm in diameter 7–13 days post-AI. The size of the CL was recorded as the mean of approximately the greatest length and width measurements. A lack of high pixel intensity associated with a young CL [59,60] was used as reference to assess CL maturity. As the presence of a central cavity is not associated with CL function [61,62,63], cavity CL were measured as solid CL. The presence of a double ovulation was established through the observation of two CL in the same ovary (unilateral bi-ovulation) or one CL in each ovary (bilateral bi-ovulation). Scanning was also performed along the dorso/lateral surface of each uterine horn for pregnancy diagnosis at 28–34 days post-AI. The presence of twins was established through the observation of two embryos within one uterine horn (unilateral twin pregnancy), or one embryo in each uterine horn (bilateral twin pregnancy). The viability of an embryo was confirmed by the observation of a heartbeat in all exams. The selected study population consisted of 752 cows, of which three failed to ovulate and one (ovulating) was culled before pregnancy diagnosis. These four cows were excluded from the study. The final study population comprised 748 ovulating cows artificially inseminated with frozen–thawed semen from six bulls by one of five technicians.

### 2.3. Genetic Merit Assessment

Genomic predictions for wellness traits and for lifetime merit are part of the herd management routine. Accordingly, ear tissue samples were obtained from all 6–10 months old calves to be submitted for genomic testing using the Clarifide Plus evaluation test (Zoetis Genetics, Kalamazoo, MI, USA) [64,65]. Samples were collected in accordance with good veterinary practices and complied with current legislation on animal welfare. Among other fertility traits, Clarifide Plus provides genomic prediction values for the risk of twin pregnancy [35]. Such values are expressed as standardized transmitting abilities (STA) with a mean of 100 and standard deviation (S.D.) of 5, and have been previously used to predict multiple ovulations and early twin pregnancies in this herd. Higher STA values are associated with a lower risk of double ovulation [45,46].

### 2.4. Data Collection and Statistical Analyses

Data collection for each single ovulating cow were parturition and AI dates; IEI before AI; milk production (mean production in the seven days before AI) (low producers < 41 kg vs. high producers ≥ 41 kg) and DIM at AI; genomic prediction values for twin pregnancy; and pregnancy 28–34 days post-AI. Additional data for double ovulating cows were number and location (right or left side) of CL 7–13 days post-AI and embryos at pregnancy diagnosis. The double ovulation rate was defined as the percentage of cows with at least two CL observed 7–13 days post-AI. AI dates were used to assess the effects of photoperiod (positive or negative) and season on the incidence of multiple ovulations and twin pregnancies. The positive photoperiod (increasing day length) extends from 22 December (sunrise 8:20 h; sunset 17:29 h) to 21 June (sunrise 5:23 h; sunset 20:35 h), whereas the negative photoperiod (decreasing day length) extends from 22 June to 21 December. The threshold for milk production was set as the median value of production recorded. In our geographical area, there are only two clearly distinguishable weather periods: warm (May to September) and cool (October to April). A clear negative effect of the warm period on ovarian activity and reproductive variables has been extensively reported [40,44,56,57]. During the cool period, temperatures < 0 °C were recorded on 32 days and temperatures > 25 °C on 7 days. During the warm period, temperatures > 25 °C were recorded on 145 days. Temperatures were collected in the shade from a meteorological station 200 m away from the study farm.

**Figure 1:**
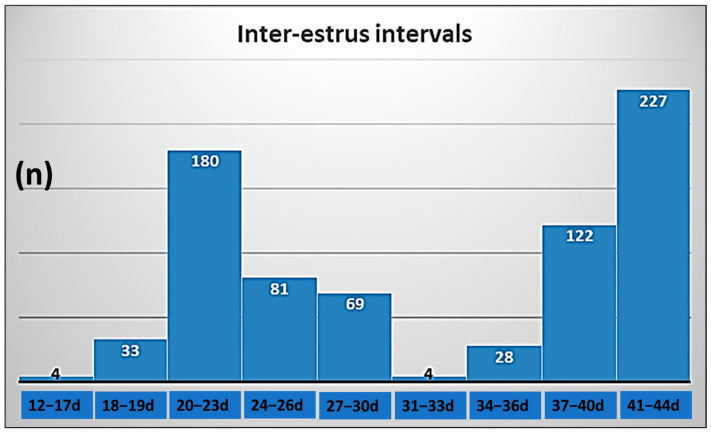
Inter-estrus intervals of cows inseminated in their second observed spontaneous estrus before Day 90 into lactation.

As IEIs ranged from 12 to 44 days (Figure 1), to examine the possible influence of photoperiod on the incidence of lengthened estrous cycles and possible relationships between IEI and double ovulations, a subset of 341 cows with IEIs from 18 to 30 days was selected. Cows were excluded from this subset if they complied with one of the three criteria: IEI shorter than 18 days (*n* = 4), IEI exceeding 30 days (*n* = 380) and IEI from 18 to 30 days including the transition period of the photoperiod, 22 June, or 21 December (*n* = 23). As in these latter cows the photoperiod was not a significant factor influencing double ovulations, they were excluded from the analyses to avoid the possible effect of day-length change on ovarian activity. The IEIs were classified as normal, from 18 to 23 days (68.9%: 235/341), or lengthened, from 24 to 30 days (31.1%: 106/341).

Three binary logistic regression analyses were performed in all cows (*n* = 748) using double ovulations, pregnancy, and twin pregnancy as the dependent variables. The factors entered in the model as independent variables were genomic prediction values and DIM as continuous variables; and season of AI (warm), photoperiod (positive), pregnancy and milk production (high production) as dichotomous variables (where “1” denotes presence and “0” absence). For the dependent variable pregnancy, double ovulations, AI technician and bull were added as factors. For the dependent variable twin pregnancy, only pregnant cows were included in the analysis. Possible interactions between milk production and season or photoperiod and between season and photoperiod were also examined.

Two further regression analyses were performed on the subset of cows with IEIs from 18 to 30 days (*n* = 341), using double ovulations and normal IEI as the dependent variables and the same independent variables used for all cows, adding IEI normal (18–23 days) as a dichotomous variable for the dependent variable double ovulation.

Regression analyses were conducted according to the Hosmer and Lemeshow method [66] using the software package PASW Statistics for Windows Version 18.0 (SPSS Inc., Chicago, IL, USA) adjusting for DIM, milk production, and time of year. Estimates and Wald 95% limits were used to calculate odds ratios (ORs) and 95% confidence intervals. Explanatory variables and interactions were assessed through a backward elimination procedure, and variables were found to significantly affect ovulation or pregnancy rate kept in the model. Significance was set at *p* < 0.05. Values are expressed as the mean ± the standard deviation (SD).

## 3. Results

Of the 748 cows enrolled, 44 (5.9%) experienced double ovulations, 323 (43.2%) were inseminated during the warm period, 343 (45.9%) during the positive photoperiod, and 466 (62.3%) became pregnant. All CL were mature and no ovulations with three or more CL were recorded at 7–13 days post-AI. Of the 466 pregnant cows, 6 (1.3%) carried twins. At least one CL ipsilateral to the embryo was observed in all pregnancies. All embryos were alive at pregnancy diagnosis. Of the 323 cows inseminated during the warm season, 193 (59.8%) became pregnant, whereas of the 343 cows inseminated during the positive photoperiod, 214 (62.4%) became pregnant. Milk production, DIM, IEI at the time of AI, and genomic STA values were 40.3 ± 5.6 (30–61) kg, 71.1 ± 8.3 (57–89) days, 31.8 ± 9.3 (12–44) days, and 101.8 ± 4.3 (87–111) units, respectively (mean ± SD; ranges between parentheses).

Table 1 summarizes the double ovulation rates, and ORs and 95% confidence intervals for all cows. The final model included the effect of genomic prediction values and photoperiod. The warm period of the year, milk production and DIM at AI proved not significant and were not included in the model. Season–milk production, photoperiod–milk production, or season-photoperiod interactions were not found. The OR for double ovulations was 0.82 (*p* < 0.0001) for each unit increase in genomic STA, and 2.3 (*p* < 0.0001) for cows inseminated during the negative photoperiod compared to those inseminated during the positive photoperiod. No factors could be associated with the dependent variable pregnancy, whereas the final model included only the effect of genomic prediction values, using the lowest value (87 units) as reference for the twin pregnancy rate (OR: 0.8; 95% confidence interval: 0.79–0.94; *p* = 0.04; *R*^2^ Nagelkerke = 0.11). No interactions were found between independent factors for these two dependent variables.

Of the subset of 341 selected cows with IEIs from 18 to 30 days, 27 (7.9%) had double ovulations, 138 (40.5%) were inseminated during the warm period, 183 (53.7%) during the positive photoperiod, and 211 (61.9%) became pregnant. Four (1.9%) of the pregnant cows carried twins. Lengthened IEIs were recorded in 106 (31.1%) cows. Milk production, DIM, IEI at the time of AI, and genomic STA values were 39.4 ± 5.4 (30–54) kg, 68 ± 6.4 (57–89) days, 22.8 ± 3.1 (18–29) days, and 101.7 ± 4.6 (88–110) units, respectively (mean ± SD; ranges between parentheses).

Table 2 summarizes the double ovulations rates, ORs and 95% confidence intervals for these cows. The final model included the effect of genomic prediction values, photoperiod, and IEI. The warm period of the year, milk production and DIM at AI proved not significant and not included in the model. Season–milk production, photoperiod–milk production, season-photoperiod, or IEI-photoperiod interactions were not found. The OR for double ovulations was 0.81 (*p* = 0.003) for each unit increase in genomic STA, 2.5 (*p* = 0.048) for cows inseminated during the negative photoperiod compared to cows inseminated during the positive photoperiod, and 2.8 (*p* = 0.02) for cows with lengthened cycles compared to cows with normal cycles.

Table 3 shows the lengthened IEI rate, OR, and 95% confidence interval for the subset of cows showing IEIs from 18 to 30 days. The final model included the effect of genomic prediction values and photoperiod. The warm period of the year, milk production, and DIM at AI proved not significant and were not included in the model. Interactions were not found. The OR for lengthened IEI was 0.91 (*p* = 0.001) for each unit increase in genomic STA, and 4.4 (*p* < 0.0001) for cows inseminated during the negative photoperiod compared to those inseminated during the positive photoperiod.

## 4. Discussion

This study has a weakness that should be considered. The number of follicular waves per estrous cycle could not be assessed under our working conditions. This means that the relationship between cycle length and the development of two or three waves discussed below is derived from previous studies.

Efforts were made to reduce variations in the general clinical condition of the cows, so that double ovulations could be attributed to factors other than health status. This is why we selected primiparous cows delivering singletons not receiving any hormone treatment before AI, and inseminated during a short postpartum interval in the first trimester of lactation. Since 1920 [55], primiparous cows have continued to show a very low twinning rate, usually less than 2% [28,39,41,42,43]. These cows are therefore considered a good model to investigate factors promoting double ovulation. With rates of 5.9% (Table 1: 44/748) or 7.9% (Table 2: 27/341) of double ovulations recorded here, factors influencing double ovulation rates other than genomic predictive values and photoperiod could be investigated. In effect, the estrous cycle length had a significant impact on the double ovulation rate.

Our study provides some of the first evidence that a lengthened estrous cycle (24–30 days) could be linked to an increased incidence of double ovulations in a dairy herd. Such long cycles will involve three or more follicular waves in contrast to the normal two-wave cycles of 18–23 days [3,5,8,9,10,19,20]. A better understanding of the mechanisms driving follicular waves may allow for improved fixed-time AI protocols [15,16,17,18]. For example, treatment with human chorionic gonadotropin five days after AI was reported to improve the fertility of cows in which the first-wave dominant follicle developed ipsilateral to the CL [53,67,68,69]. Given the high repeatability of two- or three-wave cycle patterns in each cow [20], the same repeatability would be expected in normal or lengthened cycles. Based on our IEI data, we propose the design of protocols for fixed-time AI that will induce ovulation of the dominant follicle of the second wave and so carry a very low risk of double ovulation [10,49].

As expected, the negative photoperiod was associated with greater lengthened IEI rates (48.7%) compared to the positive photoperiod (15.8%). Surprisingly, genomic prediction values were linked to lengthened estrous cycles, with an OR of 0.91 for each unit increase in genomic STA. Genomic prediction values for twin pregnancies are derived from twin calvings or advanced twin abortions [35] and are useful to detect animals at risk of double ovulation and early twin pregnancy at the herd level [45,46], with both these figures being much higher than the twinning rate [26,40,44]. In fact, in the present study in which only 1.3% of cows carried twins (6/466) genomic prediction values were able to determine the risk of early twin pregnancy with an OR of 0.82 (Table 1). Therefore, the possibility of an interaction effect between genomic prediction values and photoperiod on the IEI rate was addressed here. Thus, when including genomic values as a dichotomous variable (low prediction values < 100 units vs. high prediction values ≥ 100 units) in a further binary logistic regression analysis, results were practically the same as those provided in Table 3. However, the model had a worse *R*^2^ Nagelkerke (0.09) than the model including genomic prediction values as a continuous variable (*R*^2^ Nagelkerke = 0.20). Genomic prediction values–photoperiod interactions were not found. Hence, the independent variable genomic prediction value was a better predictor of lengthened estrus when considered as a continuous rather than dichotomous variable. Our results indicate that genomic prediction values for twin pregnancies may be associated with the risk of lengthened estrous cycle as an additional factor favoring double ovulation.

Considering all cows included in our study, no factor could be associated with the conception rate recorded. It is likely that the very high pregnancy rate (62.3%: 466/748) masked the possible influence of factors on fertility. Effectively, multiple ovulations have been often related to an increased pregnancy rate [70,71,72,73], whereas here double ovulations were not, probably due to its low incidence (5.9%: 44/748). The warm period of the year also emerged here as having no effect on the double ovulation rate. Notwithstanding, the pregnancy rate in the same herd in response to all first AIs was 47.9% during the study period. In a prior study in this herd in which 15,732 AIs over five years in cows in their third lactation or more were examined, conception rates were 30.4% and 37.4% for the warm and cool periods, respectively [40]. Efficient cooling systems may be a reason for the high fertility rate recorded in primiparous cows. In addition, the use of sex-sorted semen in heifers will favor the process of parturition and uterine involution. Parturition of a female calf leads to a subsequent smaller uterus compared to a male calf [74]. Primiparous cows already have a smaller uterus than their pluriparous partners [75,76,77], with small uterine size being associated with increased fertility [78,79,80].

The warm period, which has a great negative impact on reproductive variables in our geographical area [40,44,56,57], had no effect on any of the factors examined here. This means independence of the influence of photoperiod or genetic prediction values on the incidence of double ovulations and prolonged estrous cycles. Our data indirectly support those reported in a study of the repeatability of 2-wave and 3-wave patterns of follicular development whereby season did not influence these patterns [20].

Although the assessment of estrous detection efficiency in the herd was not an objective of the present study, IEIs observed in 380 (50.8%) cows would be considered double [21,22], most of which being between 37 and 44 days (Figure 1). This means a significant failure to detect estrus in the herd, at least in this group of cows.

## 5. Conclusions

By way of overall conclusion, double ovulations were explained by lengthened estrous cycles. Additionally, lengthened cycles and double ovulations were favored by the negative photoperiod. These findings reflect the remnants of seasonality in cattle before domestication, favoring spring-summer parturitions during times of increased food availability [81,82].

## Figures and Tables

**Table 1:** Odds ratios for the double ovulation rate variables included in the final logistic regression model (*n* = 748 ovulating primiparous cows).

Factor	Class	*n*	Double Ovulation	Odds Ratio	95% Confidence Interval	*p*
ZTWINS ^(a)^	Continuous	44/748	5.9%	0.82	0.78–0.91	<0.0001
Photoperiod ^(b)^	Positive	14/343	4.1%	Reference		
	Negative	30/405	7.4%	2.3	1.4–3.7	<0.0001

*R*^2^ Nagelkerke = 0.18. ^(a)^ Genomic prediction values for twin pregnancy. The lowest value (87 units) was used as reference. ^(b)^ Positive photoperiod (increasing day length): AI from 22 December to 21 June; negative photoperiod (decreasing day length): AI from 22 June to 21 December.

**Table 2:** Associations between double ovulations and factors included in the final logistic regression model for the subset of cows with inter-estrus intervals from 18 to 30 days (*n* = 341).

Factor	Class	*n*	Double Ovulation	Odds Ratio	95% Confidence Interval	*p*
ZTWINS ^(a)^	Continuous	27/341	7.9%	0.81	0.77–0.94	0.003
Photoperiod ^(b)^	Positive	7/183	3.8%	Reference		
	Negative	20/158	12.7%	2.5	1.01–6.6	0.048
Inter-estrus interval	Normal (≤23 d)	12/235	5.1%	Reference		
	Long (>23 d)	15/106	14.2%	2.8	1.1–7.0	0.02

*R*^2^ Nagelkerke = 0.10. ^(a)^ Genomic prediction values for twin pregnancy. The lowest value (88 units) was used as reference. ^(b)^ Positive photoperiod (increasing day length): AI from 22 December to 21 June; negative photoperiod (decreasing day length): AI from 22 June to 21 December.

**Table 3:** Odds ratios for the lengthened inter-estrus interval (>23 days) rate variables included in the final logistic regression model for the subset of cows showing inter-estrus intervals from 18 to 30 days (*n* = 341).

Factor	Class	*n*	Lengthened Interval	Odds Ratio	95% Confidence Interval	*p*
ZTWINS ^(a)^	Continuous	106/341	31.1%	0.91	0.82–1.0	0.001
Photoperiod ^(b)^	Positive	29/183	15.8%	Reference		
	Negative	77/158	48.7%	4.4	2.7–7.0	<0.0001

*R*^2^ Nagelkerke = 0.20. ^(a)^ Genomic prediction values for twin pregnancy. The lowest value (88 units) was used as reference. ^(b)^ Positive photoperiod (increasing day length): AI from 22 December to 21 June; negative photoperiod (decreasing day length): AI from 22 June to 21 December.

## Data Availability

Data available on request due to restrictions (privacy). The data are not publicly available due to privacy and third-party agreements.
